# Synthesis and cellular evaluation of click-chemistry probes to study the biological effects of alpha, beta-unsaturated carbonyls

**DOI:** 10.1016/j.redox.2022.102299

**Published:** 2022-03-23

**Authors:** Chiara Morozzi, Max Sauerland, Luke F. Gamon, Asmita Manandhar, Trond Ulven, Michael J. Davies

**Affiliations:** a Department of Biomedical Sciences, Panum Institute, University of Copenhagen, Copenhagen, 2200, Denmark; b Department of Drug Design and Pharmacology, Jagtvej 162, University of Copenhagen, Copenhagen, 2100, Denmark

**Keywords:** Dimethylfumarate, Unsaturated carbonyls, Michael adduct, Click chemistry, GAPDH, Electrophile, Keap-1, ABuCs, alpha, beta-unsaturated carbonyls, ACN, acetonitrile, DMF, dimethylformamide, DMFU, dimethylfumarate, DMSO, dimethylsulfoxide, EDC, *N*-(3-dimethylaminopropyl)-*N*′-ethylcarbodiimide, HCASMC, human coronary artery smooth muscle cells, MMFU, monomethylfumarate, TFA, trifluoroacetic acid

## Abstract

Humans are commonly exposed to α,β-unsaturated carbonyls as both environmental toxins (e.g. acrolein) and therapeutic drugs (e.g. dimethylfumarate, DMFU, a front-line drug for the treatment of multiple sclerosis and psoriasis). These compounds undergo rapid Michael addition reactions with amine, imidazole and thiol groups on biological targets, with reaction at protein Cys residues being a major reaction pathway. However, the cellular targets of these species (the ‘adductome’) are poorly understood due to the absence of readily identifiable tags or reporter groups (chromophores/fluorophores or antigens) on many α,β-unsaturated carbonyls. Here we report a ‘proof of concept’ study in which we synthesize novel α,β-unsaturated carbonyls containing an alkyne function introduced at remote sites on the α,β-unsaturated carbonyl compounds (e.g. one of the methyl groups of dimethylfumarate). The presence of this tag allows ‘click-chemistry’ to be used to visualize, isolate, enrich and characterize the cellular targets of such compounds. The probes show similar selectivity and reactivity to the parent compounds, and compete for cellular targets, yielding long-lived (stable) adducts that can be visualized in intact cells (such as primary human coronary artery smooth muscle cells), and extracted and enriched for subsequent target analysis. It is shown using this approach that dimethylfumarate forms adducts with multiple intracellular targets including cytoskeletal, organelle and nuclear species, with these including the rate-limiting glycolytic enzyme, glyceraldehyde-3-phosphate dehydrogenase (GAPDH). This approach should be amenable to use with multiple α,β-unsaturated carbonyls and a wide variety of targets containing nucleophilic sites.

## Highlights

• Humans are widely exposed to α,β-unsaturated carbonyls via drugs and environmental toxins.
• These compounds react with cellular targets, and particularly Cys residues, via Michael addition.
• Alkyne tagged derivatives have been synthesized to allow click chemistry detection.
• These tags allow visualization, extraction, enrichment and identification of adducted proteins.
• GAPDH reacts with dimethylfumarate, with adducts detected in both the cytosol and nucleus.

## Graphical abstract

**Figure:**
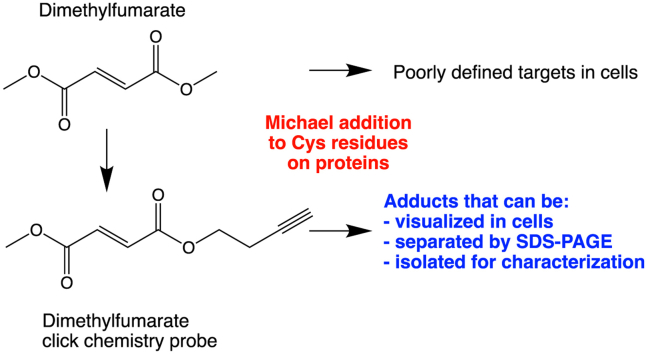

## 1 Introduction

Alpha, beta-unsaturated carbonyls (ABuCs) are abundant in natural products and synthetic versions are widely used in agricultural and industrial processes resulting in widespread human exposure [1,2]. Acrolein (2-propenal, CH_2_=CHCHO), the prototypic member of the family, is a combustion product of many organic materials (e.g. automobile exhausts, oils heated to high temperatures, cigarette smoke), and is a key intermediate in acrylic acid production. It is a powerful irritant, highly toxic and a suspect carcinogen [1,3]. Acrolein and other ABuCs (e.g. crotonaldehyde, 4-hydroxy-2-nonenal and prostaglandins PGA1/2, PGB1/2, PGJ2) are generated, *in vivo,* from polyunsaturated fatty acids either enzymatically or via radical-mediated lipid peroxidation. Some of these compounds have established signalling functions (prostaglandins) and can induce adaptive or protective responses [4,5]. Other ABuCs are pharmaceutically important, with DMFU being a front-line multiple sclerosis and psoriasis treatment, and one of the top 20 best-selling drugs [6,7]. DMFU is also reported to have immuno-modulatory, cytoprotective, antioxidant, and antitumoral effects [8], and therefore this compound in under extensive clinical evaluation for multiple conditions. The mechanism(s) of action of DMFU and other ABuCs are still poorly understood and elucidation of their cellular targets would both support their potential therapeutic application (e.g. in cancer and atherosclerosis [8,9]), and help elucidate potential toxic effects. After oral dosing, DMFU is hydrolyzed to its monomethyl derivative (MMFU) by esterases in the upper intestine, however DMFU is known to be rapidly absorbed due to its high lipophilicity and the biological actions of this drug may involve both the mono- and di-methylated species [10].

**Fig. 1:**
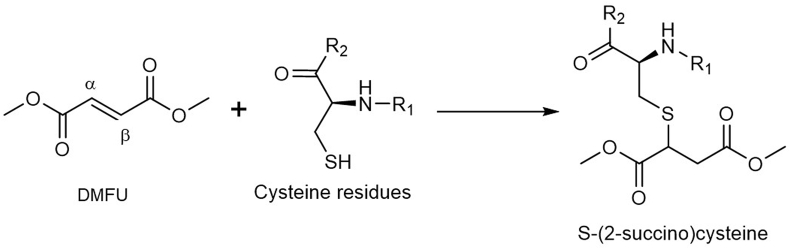
DMFU reacts with cysteine (Cys) residues on proteins via Michael addition to give S-(2-succino)cysteine adducts.

Previous kinetic and product analysis studies have identified cysteine (Cys) residues on proteins as major targets for this family of ABuCs, with adduction to Cys occurring via Michael addition reactions (Fig. 1). The kinetics of adduction depends on multiple factors including the Cys pKa, steric effects and environment [11]. A number of potential peptide and protein targets of DMFU have been identified, including hydroxycarboxylic acid receptor 2 [12], GSH [13], protein kinase Cθ [14] IRAK4 [15], GAPDH [16], and Keap1 [17,18]. However, identification of the total spectrum of protein adducts within cells, and their localization, is hampered by the low concentration of some of the adducted species, and the absence of a suitable ‘tag’ (e.g. chromophores or fluorophores, or suitable antigens and antibodies for immunoblotting studies) on the ABuC or adducted proteins. Thus, it has proved difficult to determine the exact biological targets of these species in intact (viable) cells.

To facilitate identification of cellular targets of DMFU (and other ABuCs), and potential enrichment of adduct species, we report here a proof-of-concept study, in which we describe the synthesis of click-chemistry (copper(I)-catalyzed 1,3 dipolar cycloaddition of azides to alkynes to form 1,2,3-triazoles [19,20]) probes of DMFU, and its biologically-important monomethyl hydrolysis product (monomethylfumarate, MMFU). These alkyne probes show similar chemical reactivity to the parent compounds, and have little overt toxicity at low to moderate μM concentrations. Use of these probes in primary cultures of human coronary artery smooth muscle cells has allowed detailed information to be obtained on the location of their cellular targets, via click-chemistry adduction of a fluorophore to the derivatized proteins. As the adducts are stable, they are amenable to isolation and enrichment allowing characterization of the proteins to which the tag is attached. The adducted proteins can be separated by SDS-PAGE, and readily tracked by use of the fluorescent tag; subsequent transfer to membranes and immunoblotting allows identification of cellular targets. These data indicate that cytoskeletal and nuclear proteins are prominent targets, together with glyceraldehyde-3-phosphate dehydrogenase (GAPDH).

## 2 Materials and methods

### 2.1 Reagents and syntheses

All chemicals were purchased from Sigma-Aldrich (Søborg, Denmark) and used without further purification, unless otherwise stated. Details of the synthetic procedures are given in the Supplementary data, together with ^1^H and ^13^C NMR data, HPLC traces, retention time data and mass spectroscopy data for molecular ions (Supplementary data and Supplementary Figs. 1–6). ^1^H NMR (500 MHz) and ^13^C NMR (125 MHz) spectra were recorded on a Bruker AVANCE III HD 400 MHz spectrometer at 25 °C. The deuterated solvent used was CDCl_3_ and the ^1^H spectra were calibrated using the residual proton peak at *δ* = 7.26 ppm, and ^13^C spectra via the carbon peak at *δ* = 77.36. Chemical shifts (*δ*, see Supplementary data and spectra) are reported in parts per million (ppm), and coupling constants (J) in Hertz. The following abbreviations are used in the assignment of NMR signals reported in the Supplementary data: s (singlet), d (doublet), t (triplet), td (triplet of doublets). Reactions were followed by thin-layer chromatography using precoated aluminium-backed plates, (60 F254, 0.2 mm thickness; Merck, Søborg, Denmark), with spots visualized via potassium permanganate solution, and by UPLC, using a Shimadzu Nexera UHPLC system (Shimadzu, South Rydalmere, NSW, Australia) and a Kinetex 2.6 μm EVO C18 100A Column (250 × 2.1 mm) at 40 °C. The column was eluted using a binary gradient with solvent A (0.1% v/v TFA in H_2_O) and solvent B (0.1% TFA in 80% ACN). Elution was initiated with 0% solvent B for 2 min followed by a linear gradient elution from 0 to 100% B over 7 min, 100% B for 3 min, and then re-equilibration at 0% B for 4 min. The eluted species were detected by their UV absorbance at 220 nm, with peak areas determined using Lab Solutions 5.32 SP1 software (Shimadzu). HPLC was used to confirm the purity of the final compounds. All compounds tested had a purity of ≥95% as determined by HPLC at 220 and 254 nm.

### 2.2 Cell culture and imaging

Growth media for primary human coronary artery cells (212–500) was purchased from tebu-bio (Roskilde, Denmark). PBS (14190169), Alexa488-azide (A10266) and DAPI (D3571) were bought from Thermo Fisher Scientific (Søborg, Denmark). Trypsin-EDTA (T4299) was from Sigma Aldrich. Chambered cover glass slides (734–2061), paraformaldehyde (J61899.AK), Triton x-100 (21123), mounting media (101098–042), ascorbic acid (1.00468.0100) and CuSO_4_ (84845.230) were obtained from VWR (Søborg, Denmark). Human coronary artery smooth muscle cells (HCASMC, donor #1522, from tebu-bio) were cultured until confluent in smooth muscle cell growth medium at 37 °C and 5% CO_2_. The cells were then detached using trypsin-EDTA for 5 min. The cell numbers were adjusted to 50,000 cells mL^−1^, with 500 μL of cell suspension placed in each well, in chambered cover-glass wells, and incubated for 36 h. The cells were then incubated with DMFU (0–90 μM) for 2 h followed by 15 min incubation with DMFU probe (30 μM). Alternatively, cells were pre-incubated with MMFU for 20 h (0–250 μM) followed by a 4 h incubation with MMFU probe (40 μM). The succinate-derived probe, which did not give rise to any fluorescent labeling, was used as a negative control.

The cells were washed with PBS and fixed in 4% paraformaldehyde for 15 min. Permeabilization was achieved using 0.1% Triton in PBS for 10 min. The cells were then covered with 100 μL of the ‘click chemistry’ reaction mixture containing 0.1 M phosphate buffer (pH 7.4), 2 mM CuSO_4_, 20 mg mL^−1^ ascorbic acid, and 3 μM of azide-functionalized Alexa488. The cells were then incubated for 10 min at 21 °C. Nuclei were stained with 4′,6-diamidino-2-phenylindole (DAPI, 10 μg mL^−1^) for 10 min at 21 °C. Cells were then washed and covered with 100 μL of Vectra shield mounting media. Cells were imaged using a LSM780 confocal microscope using Zen black software and analyzed using a custom-written Python script (see Supplementary Data).

### 2.3 Cell viability

Human coronary artery smooth muscle cells (5 × 10^4^) were placed in a 24-well plate and allowed to adhere overnight. The cells in each well were then treated with 30 μM of DMFU-, MMFU- or succinate probe for 15 min (DMFU-probe) or 4 h (MMFU-, succinate-probes). The cell viability was then determined by quantifying the activity of the intracellular enzyme lactate dehydrogenase (LDH) that is released into the supernatant arising from cell damage, with the LDH activity in the supernatant compared to the total LDH activity (cell plus supernatant). Thus, the supernatant (500 μL) was collected and stored on ice, and the cells then washed 3 times with PBS before being lysed in 500 μL water. The LDH activity in both samples were measured by adding 200 μL of 0.225 mM NADH and 2.5 mM sodium pyruvate to 10 μL of the sample. NADH consumption was quantified using a Clariostar Plate reader via measurement of the absorbance at 340 nm over a period of 30 min. The cell viability was then calculated as the LDH activity of the supernatant divided by the combined LDH activity of supernatant and lysate expressed as a percentage. The data presented are from three independent experiments. The percentage activity was normalized to the untreated controls.

### 2.4 SDS-PAGE and immunoblotting analyses

Human coronary artery smooth muscle cells were seeded in 6 well plates, cultured until confluent, then treated with 100 μM DMFU-, MMFU-, or succinate-probes for 4 (DMFU-probe) or 24 h (MMFU- and succinate-probes). The cells were then washed, removed from the plates and pelleted by centrifugation. The cells were lysed in 40 μL 4% SDS, then incubated with 1% TFA for 30 min to hydrolyze DNA. After neutralization (150 μL 0.1 M TRIS base), 50 μL of lysate was subjected to click chemistry using 1.1 mM CuSO_4_, 17 mM ascorbate, 8.5 mM Alexa 488-Azide, 3 mM hydroxypropyltriazolylmethylamine, in 5% DMSO and 17 mM aminoguanidine hydrochloride for 2 h at 21 °C. The samples were then incubated with 4 μL SP3 Beads (50 mg mL^−1^) in 70% ACN for 20 min to allow protein binding to the beads. The beads were then washed sequentially with 70% ethanol and 100% ACN. Proteins were dissolved in 1x LDS Sample buffer (NP0007) with 1x reducing agent (NP0009) (15 min, 60 °C), loaded and separated by SDS-PAGE on 4–12% Bis-Tris gels (NP0322BOX). The gels were run for 40 min at a constant 170 V using MES running buffer (NP0002) and subsequently imaged using a Sapphire Biomolecular Imager. The proteins were then transferred to a PVDF membrane (IB24002) and stained using anti-GAPDH primary antibody (GA1R) and a fluorescently-coupled secondary antibody (Azure 800). Images were then captured using a Sapphire Biomolecular Imager, and are presented as representative data obtained from at least three independent experiments.

### 2.5 Chromatographic purification of samples and LC-MS analyses

Chromatography purifications were performed through HPLC with the sample being loaded as a concentrated solution in the same eluent. The column was eluted using a gradient method with solvent A (0.1% v/v TFA in H_2_O) and solvent B (0.1% TFA in 90% ACN). Fractions containing the product were identified, combined, and the solvent was removed in vacuo. Low-resolution mass spectrometry and LC-MS analyses were performed on a Bruker Daltonics MicroToF-LC instrument (atmospheric pressure ionization, electrospray mass spectroscopy) in either the positive- or negative-ion mode. MS parameters were optimized to the following: set capillary 4500 V, set end plate offset −500 V, set nebulizer 1.0 Bar, set dry heater 200 °C, set dry gas 8.8 L min^−1^, set divert valve source. Full scan mass spectra were recorded over a range of *m/z* 50–3000.

### 2.6 Immunocytochemistry

Cells were treated and subjected to click chemistry adduction as described in section 2.4. The treated HCASMC were then blocked with 3% BSA (Sigma, A9647-100G) in Tris-buffed saline containing Tween 20 (TBST), for 60 min at 21 °C, followed by incubation with the primary anti-GAPDH antibody (diluted 1:100; Thermofisher, GA1R) in 3% BSA in TBST overnight at 4 °C. The cells were then washed 3-times with TBST followed by the incubation with the corresponding secondary antibody (goat-anti mouse IgG, diluted 1:1000; Thermofisher, A-11032) in TBST for 60 min. Cells were then washed 3 times with TBST, and once with Tris-buffered saline, before being covered with Vectra shield mounting medium. Cell images were captured using a Zeiss LSM 780 confocal microscope with the following conditions: DAPI, *λ*_ex_ 405 nm, *λ*_em_ 454 nm; DMFU probe, *λ*_ex_ 488 nm, *λ*_em_ 525 nm; GAPDH *λ*_ex_ 595 nm, *λ*_em_ 668 nm.

### 2.7 Computational calculation of electrophilic indices

Spartan'20 v1.14 software was used to calculate the energy levels of ABuCs for the highest occupied molecular orbital (HOMO) and the lowest unoccupied molecular orbital (LUMO) for the equilibrium geometry of the ground state in water, using the Density Functional B3LYP 6-31G* dataset. Those numbers were used to calculate the softness σ = 2/(E_LUMO_ – E_HOMO_), the chemical potential μ = (E_LUMO_ + E_HOMO_)/2 and the electrophilic index ω = 1/2 σμ^2^. The electrophilic index is reported to provide an estimate of the reactivity towards soft nucleophiles [21,22].

### 2.8 Statistics

Data analysis and statistical analyses were conducted using Graph Pad Prism 9. Data are displayed as boxplots (mean ± SD) and were compared statistically using one way ANOVA with post-hoc testing for multiple comparisons with significance, indicated by *, assumed with an adjusted p value of <0.05.

## 3 Results and discussion

### 3.1 Synthesis of alkyne-tagged analogues of DMFU, MMFU and succinate

**Fig. 2:**
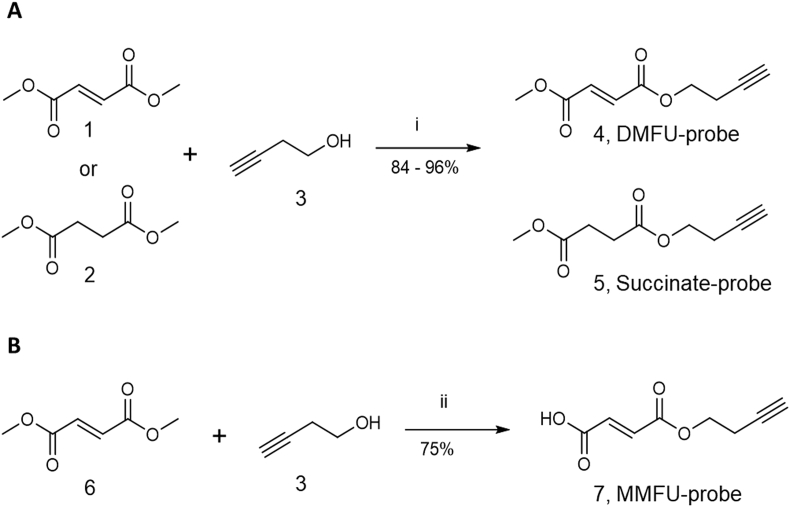
Summary of synthetic approaches to give (A) the DMFU (4) and succinate (5) probes, and (B) the MMFU-probe (7) using esterification with but-3-yn-1-ol (3). Reaction reagents and conditions: i) *N*-(3-dimethylaminopropyl)-*N*′-ethylcarbodiimide hydrochloride and 4-(dimethylamino)pyridine in ACN at 45 °C for 45 min. ii) *N*-(3-dimethylaminopropyl)-*N*′-ethylcarbodiimide hydrochloride and N-methylmorpholine in dry DMF, initially at 0 °C then allowed to warm up to 21 °C, overnight. For detailed descriptions of the procedures and reagents see Supplementary data.

DMFU and MMFU, together with succinate as a negative control which is incapable of Michael addition due to the absence of the double bond, were chemically modified by replacement of one of the methyl groups in each parent compound with a butynyl-tag, using but-3-yn-1-ol (3) (Fig. 2). This yielded the corresponding probes DMFU-probe (4), MMFU-probe (7), and succinate-probe (5). Monomethylfumarate and monomethyl hydrogen succinate were esterified with but-3-yn-1-ol (3) to give the DMFU (4) and succinate (5) probes in yields of 84% and 96% respectively. A variation on this route was used to synthesize the MMFU-probe (7), with fumaric acid (6) selectively mono-esterified with (3) at one of the carboxylate groups, using *N*-(3-dimethylaminopropyl)-*N*′-ethylcarbodiimide hydrochloride and N-methylmorpholine in DMF. Each probe was fully characterized (see Supplementary Data) before use in cell studies.

### 3.2 Effects of alkyne probes on cell viability

**Fig. 3:**
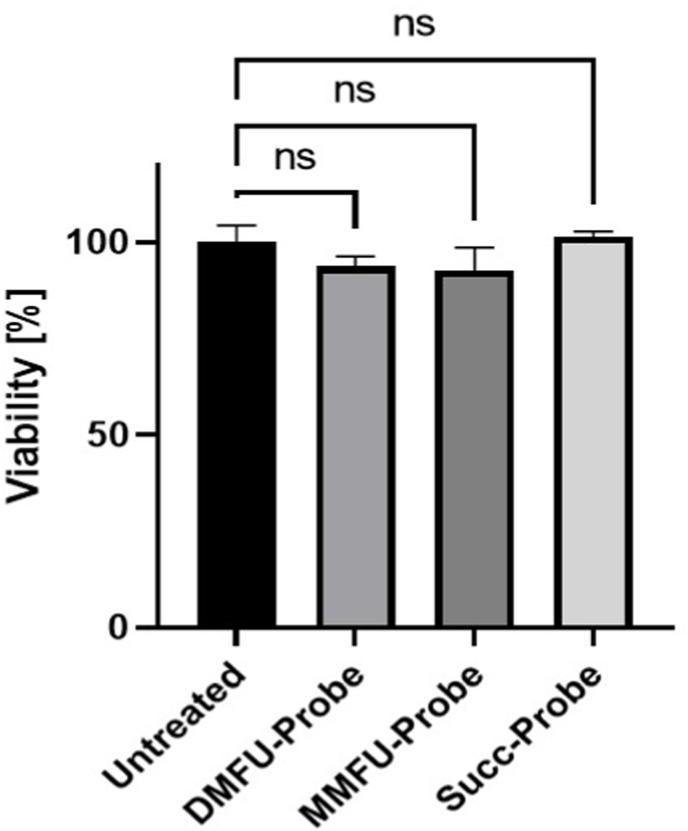
Human coronary artery smooth muscle cells (HCASMC) were seeded into 24 well plates and cultured until confluent. Each well was then treated with 30 μM of DMFU (for 15 min), MMFU or succinate probe (for 4 h). After the incubation, the supernatant (500 μL) was collected, and the cells lysed in water (500 μL). Aliquots (10 μL) of each sample were then monitored over 30 min at 340 nm to quantify loss of NADH using the lactate dehydrogenase assay (see Materials and methods). Data were normalized to the untreated samples (taken as 100%) and are presented as % viability relative to the controls, as mean values +SD. No statistically-significant differences (p > 0.05) were determined between the conditions using Graphpad Prism 9 and one-way ANOVA, with testing for multiple comparisons.

In order to determine whether the alkyne-tagged probes showed significant cellular toxicity which would prohibit biological use, primary human coronary artery smooth muscle cells (HCASMC), a key cell type in the development and progression of cardiovascular disease, were treated with 30 μM DMFU or MMFU probe for 15 min to allow target adduction. These reaction conditions did not alter cell viability, with this being >90% as determined by the extent of release of lactate dehydrogenase from the cells into the media (Fig. 3).

### 3.3 Visualization of probe adducts in HCASMC by click-chemistry fluorescent tagging

**Fig. 4:**
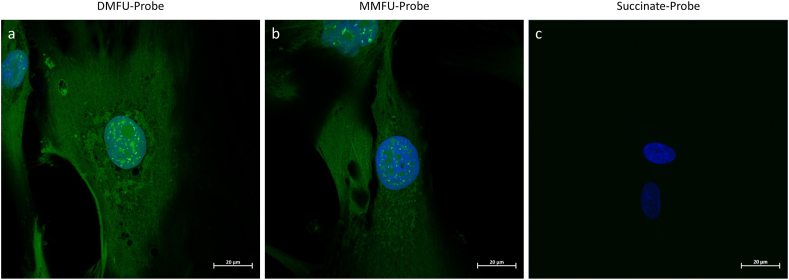
High resolution images of human coronary artery smooth muscle cells (5 × 10^4^ cells per well) treated with 30 μM of (a) DMFU probe (4), (b) MMFU probe (7), and (c) succinate probe (5) in growth media for 15 min (a), or 4 h (b, c) respectively. Cells were then fixed, permeabilized and then coupled to azide-derivatized Alexa 488 (3 μM) in presence of CuSO_4_ (2 mM) and ascorbic acid (100 mM) (see Materials and methods). Fluorescent images of the adducted species were obtained by confocal microscopy (green fluorescence, *λ*_ex_ 488 nm, *λ*_em_ 543 nm). Nuclei were counter-imaged with DAPI (blue). Scale bars 20 μm. (For interpretation of the references to colour in this figure legend, the reader is referred to the Web version of this article.)

Probe-loaded HCASMC (see above) were incubated with a commercial azide-derivatized Alexa 488 fluorophore under click-chemistry conditions (see Materials and methods), to attach the fluorophore to the DMFU/MMFU adducted species or cells loaded with the (negative control) succinate probe. The resultant cultures were subsequently subjected to fluorescent imaging (Fig. 4). DAPI was used to delineate cell nuclei.

The resulting data show similar patterns of fluorescence throughout the cell cytosol and nucleus for both the DMFU- and MMFU-probes, consistent with adduction of the probe species to multiple cellular proteins. The similarity of the pattern of fluorescence between the DMFU- and MMFU-probes suggests that the specificities of these two probe species are similar, though a significantly lower intensity of fluorescence was detected for the MMFU probe, when compared to the DMFU probe. This is consistent with the reported lower reactivity of MMFU when compared to DMFU [16,23].

This lower reactivity does not correlate well with computational calculations of the electrophilic index for the probes, and their parent compounds, which all yielded similar values (electrophilic index values: DMFU, 4.59; DMFU probe, 4.66; MMFU, 4.70; MMFU probe, 4.74). These values were also greater than those calculated for other ABuC family members (acrolein, 3.57; crotonaldehyde, 3.28; 4-hydroxynonenal, 3.32; cyclopentenone, 2.82) and the control compound, succinate (1.82). In contrast, experimental studies in which rate constants were determined for reaction of many of these species with thiols, indicates that DMFU is markedly less reactive than other members of the family [11]. However, these experimental data indicate that multiple other factors, including the pK_a_ of the attacking thiol, and steric and electronic factors, also play a key role in determining the overall rate and extent of reaction [11].

With both the DMFU and MMFU probes, the fluorescent images indicate that multiple targets are subject to adduction by the probes, rather than single species, with widespread fluorescence detected in the cytosol, nucleus, subcellular organelles and at the plasma membrane. This conclusion is supported by the subsequent isolation and separation of multiple adducted proteins (see below). Of particular note, is the widespread fibre-like patterning of the cytosol, which is consistent with binding to cytoskeletal components, the presence of areas of high fluorescence intensity at discrete locations surrounding the nucleus, consistent with sub-cellular organelle localization, and intriguingly, the presence of punctate staining (‘dots’) within the cell nuclei. The latter is ascribed to probe adduction to specific nuclear proteins. This last observation is consistent with the role of DMFU and MMFU is modulating gene expression [24].

**Fig. 5:**
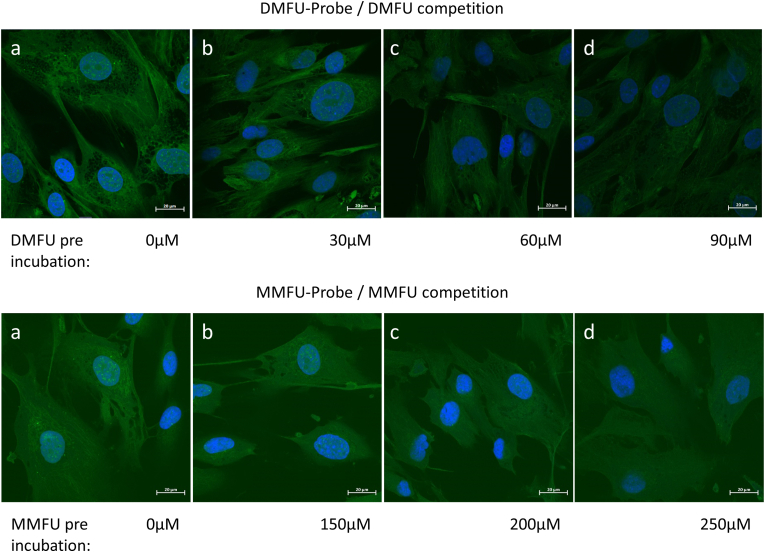
(a) Human coronary artery smooth muscle cells (5 × 10^4^ cells per well) were treated with (top row) 30 μM of DMFU probe (4) or (bottom row) 30 μM of MMFU probe (7), in growth media for 15 min, then coupled to azide-derivatized Alexa 488 (3 μM) before acquisition of fluorescent images of the adducted species (green fluorescence, *λ*_ex_ 488 nm, *λ*_em_ 543 nm). Nuclei were counter-imaged with DAPI (blue). (b–d) Cells were pre-incubated with (top row) 30–90 μM parent DMFU for 2 h or (bottom row) 150–200 μM of MMFU for 4 h, as a competitor for the cellular targets before reaction with the DMFU or MMFU probes. (For interpretation of the references to colour in this figure legend, the reader is referred to the Web version of this article.)

**Fig. 6:**
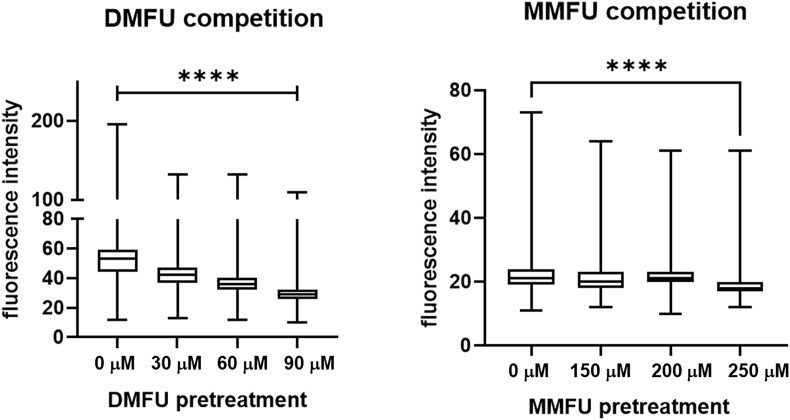
HCASMC were seeded onto glass cover slips and treated with DMFU-probe and MMFU-probe as described in the Materials and methods. Subsequently Alexa azide-488 was added to the cells to fluorescently-label the probe-modified proteins. Pretreatment with DMFU (left panel) or MMFU (right panel) lowered the fluorescence intensity significantly, consistent with the probes having the same specificity as the parent compounds. Pixel density of fluorescence emission from each slide was determined using an automated analysis system and a Python script (see Supplementary data) with 220000 pixels analyzed per image. Statistical analysis was carried out using Graph Pad Prism 9, using a one-way ANOVA test with post-hoc testing for multiple comparisons. Statistical significance is indicated by **** at the p < 0.001 level.

To confirm that the alkyne function, which replaces one of the methyl groups of the parent, does not perturb target selectivity, HCASMC were pre-treated with the respective parent compound (DMFU or MMFU), at varying concentrations (up to 250 μM) for periods of up to 20 h, before addition of the respective probes to investigate whether there is competition for the same targets. Pre-treatment of HCASMC with DMFU for 2 h before DMFU probe addition decreased the fluorescent signal from the probe species in a dose dependent manner, as determined visually (Fig. 5), or by densitometric analysis of the fluorescence from the cultures (Fig. 6). In the latter case, an automated Python script was developed (Supplementary data) that allowed quantification of up to 220000 pixels per image allowing ready quantification and analysis of changes. The observed differences are consistent with competition between DMFU and the DMFU probe for available targets, and indicates that the alkyne tag does not interfere with the gross selectivity of reaction of the probe versus parent for cellular targets. Pre-treatment of HCASMC with up to 250 μM MMFU for up to 20 h showed a smaller effect on the fluorescent intensity consistent with the slower rate of target adduction by MMFU, compared to DMFU [16].

**Fig. 7:**
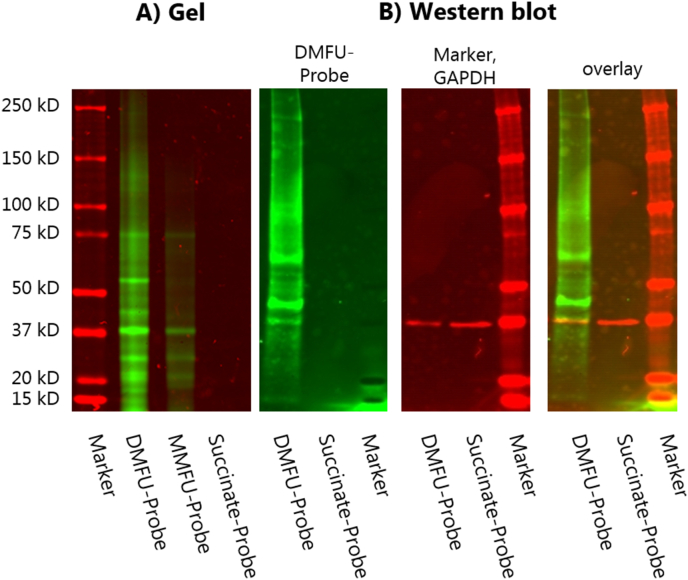
HCASMC were seeded in 6 well plates, cultured until confluent, then treated with 100 μM DMFU-, MMFU- or succinate-probes for 4 (DMFU-probe) or 24 h (MMFU-, succinate-probes). The cells were then subjected to ‘click chemistry’ conditions and processed as described in the Materials and methods to give fluorescently-tagged species. These were then isolated from the cultures using SP3 beads, then eluted. The tagged proteins were separated by SDS-PAGE and then imaged directly for fluorescence (panel A), or (panel B) transferred to a PVDF membrane and analyzed for the presence of the probe, immunoblotted using an anti-GAPDH primary antibody (clone GA1R) and a fluorescent secondary antibody (Azure 800). The image at the righthand side presents an overlay image of fluorescence and immunoblot data showing the co-localization of the band from GAPDH. Molecular mass markers were included as indicated.

**Fig. 8:**
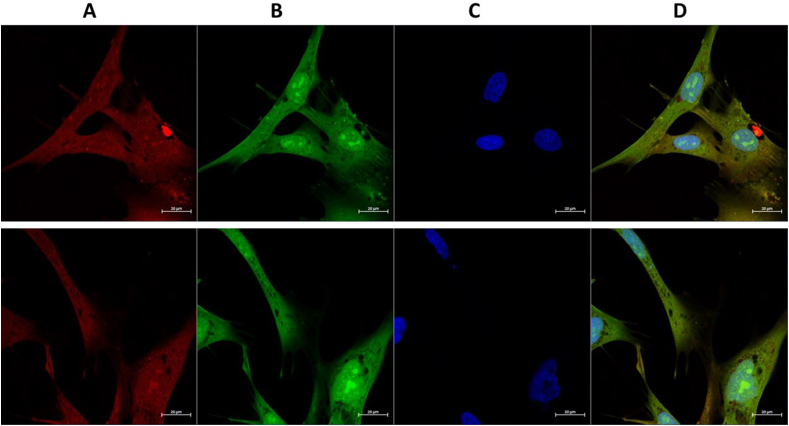
HCASMC were seeded in 6 well plates, cultured until confluent, then treated with 100 μM DMFU-probe for 4 h. The cells were then subjected to ‘click chemistry’ conditions and processed as described in the Materials and methods to give fluorescently-tagged species or DAPI-stained species. The cells were then either imaged for fluorescence directly (DMFU probe and DAPI), or by using an anti-GAPDH primary antibody and a fluorescently-tagged secondary antibody as described in the Materials and methods. The top and bottom rows of images are representative data from independent experiments from a total of three. Panel A: cells probed with anti-GAPDH antibody; panel B: cells imaged directly for protein-bound DMFU-probe; panel C: cells stained with DAPI to delineate cell nuclei; panel D: composite merged image from combined panels A–C.

The adducted species showed high stability (detectable for 2 weeks after initial formation in paraformaldehyde-fixed cells), consistent with irreversible adduction of the ABuC to the targets, and an absence of rapid turnover or repair of the modified species. This high stability allows subsequent extraction of the adduct species (with attached fluorophore tag) for further characterization. As shown in Fig. 7A, the tagged proteins were stable to separation by SDS-PAGE, with the intensity of fluorescence detected for the three probes reflecting the order of reactivity of the probes with biological targets (i.e. DMFU-probe > MMFU-probe » succinate probe). The detection of multiple specific bands from the samples treated with the DMFU- and MMFU-probes is consistent with multiple discrete targets within the cells, with most of the bands detected with the MMFU-probe reflecting those detected with the DMFU-probe, albeit at much lower intensity. However, some species appear to be preferentially (or more heavily) modified by the DMFU-probe, over the MMFU-probe, with a greater number of bands detected in the former case (cf. additional bands for the former at ∼120, ∼55, ∼35 and ∼15–18 kDa). No bands were observed for the succinate probe consistent with a lack of adduction of this species (as expected from the absence of the double bond), and an absence of non-specific binding. Transfer of the tagged proteins to PVDF membranes and subsequent immunoblotting showed the presence of the key intracellular glycolytic protein glyceraldehyde-3-phosphate dehydrogenase (GAPDH) in all the samples (Fig. 7B, red bands) with this band co-eluting with a band tagged with the DMFU-probe (green bands) indicating that GAPDH is a major intracellular target for DMFU (cf. overlay image in Fig. 7B). This is consistent with data indicating that this enzyme is a target for DMFU [16], with adduction likely to occur at the (low pK_a_) active site Cys152 residue. Whether the effects of DMFU are solely on GAPDH glycolytic activity or also modulate its effects on transcription activation, initiation of apoptosis or intracellular protein trafficking remain to be established [25,26]. Efforts to identify the other proteins that give rise to fluorescent bands in Fig. 7A are currently underway. Confirmation of the targeting of GAPDH by the DMFU probe was obtained from immunocytochemistry of the treated HCASMC. The images obtained show clear co-localization of GAPDH and the probe (Fig. 8), both within the cell cytosol and nuclei, consistent with the dual role of GAPDH as both a key enzyme in glycolysis, and also as a signalling agent that translocates to the nucleus, where it is associated with nuclear complexes and binds to telomeres [[27], [28], [29]].

## 4 Conclusions

The data reported here indicate that it is possible to visualize adducts of α,β-unsaturated carbonyls to biological molecules within intact viable cells by the use of analogues with alkyne tags present at sites remote from the reaction site. The subsequent use of ‘click-chemistry’ to derivatize the alkyne after adduction allows the proteins targeted by these species to be imaged, isolated, enriched and identified. The data obtained indicate that the alkyne tag does not markedly affect target specificity, and that the click-chemistry adducts are stable to further analysis (e.g. extraction and characterization). We envisage that alternative click-chemistry ‘handles’, such as biotin tags, will allow pull-down and enrichment of adducts for proteomic analysis. Furthermore, we believe that this approach will be applicable to a wide range of drugs and environmental agents with similar α,β-unsaturated carbonyl (and other) functional groups. A similar protocol should also allow adducts formed on other biological macromolecules (e.g. DNA, RNA, lipids etc) to be identified, as the click-chemistry addition of the detection agent (e.g. fluorophore) should not be markedly affected by the species to which the DMFU/MMFU is attached.

## Declaration of competing interest

The authors declare the following financial interests/personal relationships which may be considered as potential competing interests:

MJD declares consultancy contracts with Novo Nordisk A/S. This funder had no role in the design of the study; in the collection, analyses, or interpretation of data; in the writing of the manuscript, or in the decision to publish the results. The other authors declare no conflict of interest.
