# Supplementary material for: Synthesis and cellular evaluation of click-chemistry probes to study the biological effects of alpha, beta-unsaturated carbonyls
Source: Redox Biol. 2022 Mar 23;52:102299. doi: 10.1016/j.redox.2022.102299 (PMC8966197; doi:10.1016/j.redox.2022.102299)
Supplement: Multimedia component 1 [file mmc1.pdf]

Synthesis and cellular evaluation of click-chemistry probes to study the biological effects of alpha, beta-unsaturated carbonyls

Chiara Morozzia [a,†], Max Sauerland [a,†], Luke F. Gamon [a], Asmita Manandhar [b], Trond Ulven [b], Michael J. Davies [a,*]

[a] *Department of Biomedical Sciences, Panum Institute, University of Copenhagen, Copenhagen 2200, Denmark*

[b] *Department of Drug Design and Pharmacology, Jagtvej 162, University of Copenhagen, Copenhagen*

2100, Denmark

SUPPLEMENTARY DATA

---

† CM and MS contributed equally to this work

* Corresponding author

E-mail address: davies@sund.ku.dk (M.J. Davies)

Abbreviations used:

ABuCs, alpha, beta-unsaturated carbonyls; ACN, acetonitrile; DMF, dimethylformamide; DMFU, dimethylfumarate; DMSO, dimethylsulfoxide; EDC, *N*-(3-dimethylaminopropyl)-*N*′-ethylcarbodiimide; HCASMC, human coronary artery smooth muscle cells; MMFU, monomethylfumarate; TFA, trifluoroacetic acid

Detailed synthetic procedures

Synthesis of but-3-yn-1-yl methyl fumarate (4)

Synthesis of this compound followed a method reported previously [1]. A solution of monomethylfumarate (1.17 mmol, 152 mg), *N*-(3-dimethylaminopropyl)-*N'*-ethylcarbodiimide hydrochloride (1.44 mmol, 276 mg), 4-(dimethylamino)pyridine (2.9 mmol, 354 mg) in ACN (15.5 mL) was reacted with 3-butyn-1-ol (1 mmol, 0.08 mL) and stirred at 45 °C for 30 min. The progress of the reaction was monitored by thin-layer chromatography until completion. The solvent was removed in vacuo and the residue was dissolved in diethyl ether (26 mL), washed with 3 M HCl (2 x 20 mL), saturated $NaHCO_3$ solution (2 x 20 mL), and brine (20 mL). The residue was concentrated in vacuo and the expected product was obtained as light orange powder in 84% yield with purity of 97%. ^{1}H-NMR (400 MHz, in $CDCl_3$): δ_H 6.88 (s, 2H, $C\underline{H}=C\underline{H}$), 4.31 (t, 2H, *J* = 8.0 Hz, $OC\underline{H_2}$), 3.81 (s, 3H, $OC\underline{H_3}$), 2.59 (td, 2H, *J* = 2.6, 6,7 Hz, $CC\underline{H_2}CH_2$), 2.01 (s, 1H, $C\underline{H}\equiv CCH_2$) ppm. ^{13}C-NMR (125 MHz, in CH_3OD): δ_C 165.47 ($\underline{C}=O$), 164.77 ($\underline{C}=O$), 133.89 ($\underline{C}H=CH$), 133.44 ($CH=\underline{C}H$), 79.73 ($CH\equiv\underline{C}CH_2$), 70.32 ($O\underline{C}H_2$), 63.07 ($O\underline{C}H_3$), 52.49 ($\underline{C}H\equiv CCH_2$), 19.06 ($C\underline{C}H_2CH_2$) ppm. HPLC: (gradient solvent A/solvent B from 100/0 to 0/100 in 15 min, flow: 1.1 mL min^{-1}, λ = 254 nm): t_R 6.23 min. MS (ES+): *m/z* 183.06 $[M+H]^+$.

Synthesis of but-3-yn-1-yl methyl succinate (5)

Synthesis of this compound followed a method reported previously [1]. A solution of monomethyl hydrogen succinate (0.6 mmol, 79 mg), *N*-(3-dimethylaminopropyl)-*N'*-ethylcarbodiimide hydrochloride (0.7 mmol, 134 mg), 4-(dimethylamino)pyridine (1.5 mmol, 183 mg) in ACN (7.8 mL) was reacted with 3-butyn-1-ol (0.5 mmol, 0.04 mL) and stirred at 45 °C for 30 min. The reaction

mixture was monitored by thin-layer chromatography until completion. The solvent was removed in vacuo and the residue was dissolved in diethyl ether (13 mL), washed with 3 M HCl (2 x 10 mL), saturated $NaHCO_3$ solution (2 x 10 mL), and brine (10 mL). The residue was concentrated in vacuo and the expected product was obtained as colorless oil in 96 % yield with purity of ~90%. ^{1}H-NMR (400 MHz, in $CDCl_3$): δ_H 4.21 (t, 2H, *J* = 8.0 Hz, OC$\underline{H_2}$), 3.70 (s, 3H, OC$\underline{H_3}$), 2.68-2.63 (m, 4H, COC$\underline{H_2}$C$\underline{H_2}$CO), 2.53 (td, 2H, *J* = 2.6, 6.8 Hz, C$\underline{H_2}$$CH_2$), 2.00 (s, 1H, C$\underline{H}$≡$CCH_2$) ppm. ^{13}C-NMR (125 MHz, in CH_3OD): δ_C 172.65 ($\underline{C}$=O), 172.01 ($\underline{C}$=O), 79.58 (CH≡$\underline{C}CH_2$), 69.90 (O$\underline{C}H_2$), 62.36 (O$\underline{C}H_3$), 29.05 (CO$\underline{C}H_2CH_2$CO), 28.08 ($COCH_2\underline{C}H_2$CO), 18.93 (C$\underline{C}H_2CH_2$) ppm. HPLC: (gradient solvent A/solvent B from 100/0 to 0/100 in 15 min, flow: 1.1 mL min^{-1}, λ = 220 nm): t_R 6.23 min. MS (ES+): *m/z* 185.07 $[M+H]^+$.

Synthesis of (*E*)-4-(but-3-yn-1-yloxy)-4-oxobut-2-enoic acid (7)

Synthesis of this compound followed a method reported previously [2]. Fumaric acid (306.5 mg, 2.64 mmol) and but-3-yn-1-ol (200 µL, 2.64 mmol) were dissolved in anhydrous DMF (5 mL). The flask was cooled down to 0 °C, and N-methylmorpholine (290 µL, 2.64 mmol) was added followed by *N*-(3-dimethylaminopropyl)-*N*'-ethylcarbodiimide hydrochloride (506.2 mg, 2.64 mmol). The reaction mixture was stirred 12 h at 20 °C. Then, 25 mL ethyl acetate was added and the solution extracted with saturated $NaHCO_3$ solution (3 x 25 mL). The aqueous layer was acidified with 1 M HCl to pH 2. The product was the extracted with ethyl acetate (3 x 25 mL). The combined organic phases were washed with 3.0 M $CaCl_2$ solution (25 mL) and dried over anhydrous $MgSO_4$, filtered, and concentrated. The crude material was then purified by preparative HPLC (0-70% solvent B in 12.5 min) to afford the desired product in 9 % yield as a white solid. ^{1}H-NMR (400 MHz, in $CDCl_3$): δ_H 10.64 (s, 1H, COO$\underline{H}$), 6.97 (d, *J* = 15.8 Hz, 1H, C$\underline{H}$=CH), 6.88 (d, *J* = 15.8 Hz, 1H, CH=C$\underline{H}$), 4.33 (t, *J*

= 6.7 Hz, 2H, OC<u>H</u>$_2$), 2.60 (td, *J* = 6.7, 2.7 Hz, 2H, CC<u>H</u>$_2$CH$_2$), δ 2.02 (t, *J* = 2.7 Hz, 1H, , C<u>H</u>≡CCH$_2$) ppm. ^{13}C-NMR (125 MHz, in CH$_3$OD): δ_C 170.1 (RO<u>C</u>=O), 164.5 (HO<u>C</u>=O), 135.4 (<u>C</u>H=CH), 133.2 (CH=<u>C</u>H), 79.6 (CH≡<u>C</u>CH$_2$), 70.4 (O<u>C</u>H$_2$), 63.3 (<u>C</u>H≡CCH$_2$), 19.0 (C<u>C</u>H$_2$CH$_2$) ppm. HPLC: (gradient solvent A/solvent B from 100/0 to 0/100 in 20 min, flow: 1.0 mL min^{-1}, λ = 254 nm): t_R 10.01 min. MS (ES+): *m/z* 167.04 [M-H]$^+$.

Supplementary Figure 1. NMR analyses of but-3-yn-1-yl methyl fumarate (**4**)

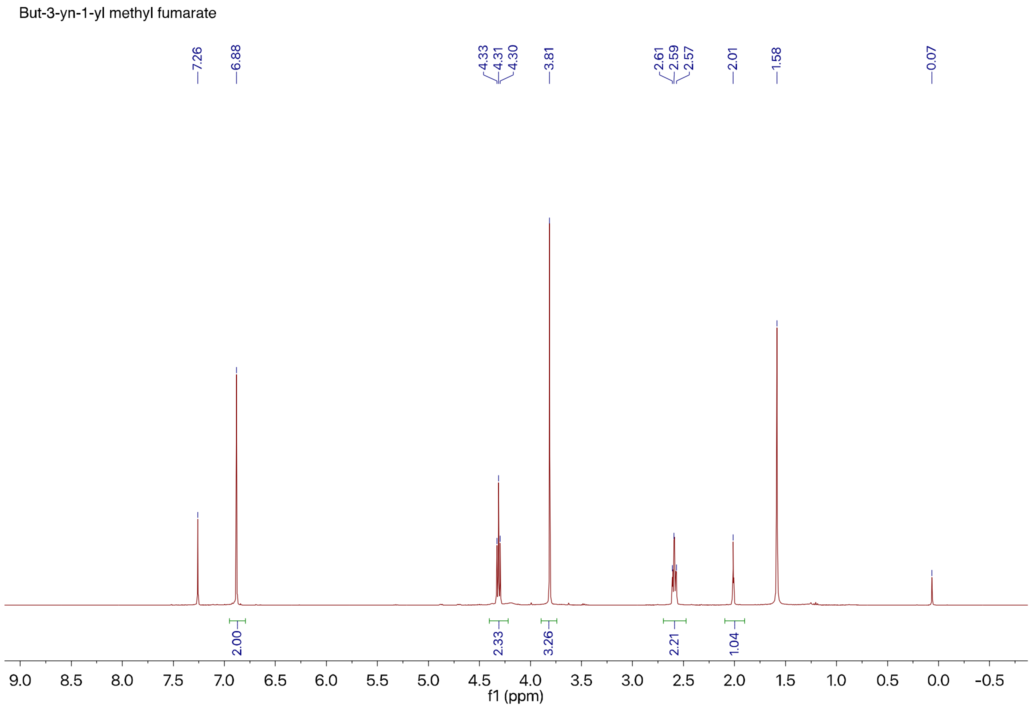

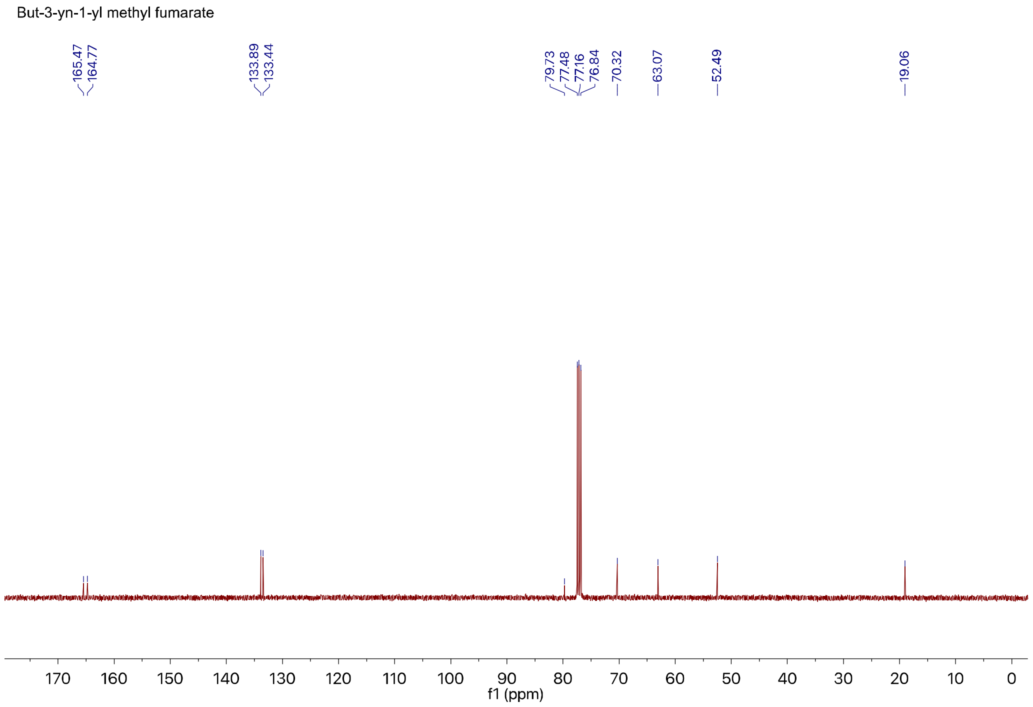

Supplementary Figure 2. HPLC analyses of but-3-yn-1-yl methyl fumarate (**4**)

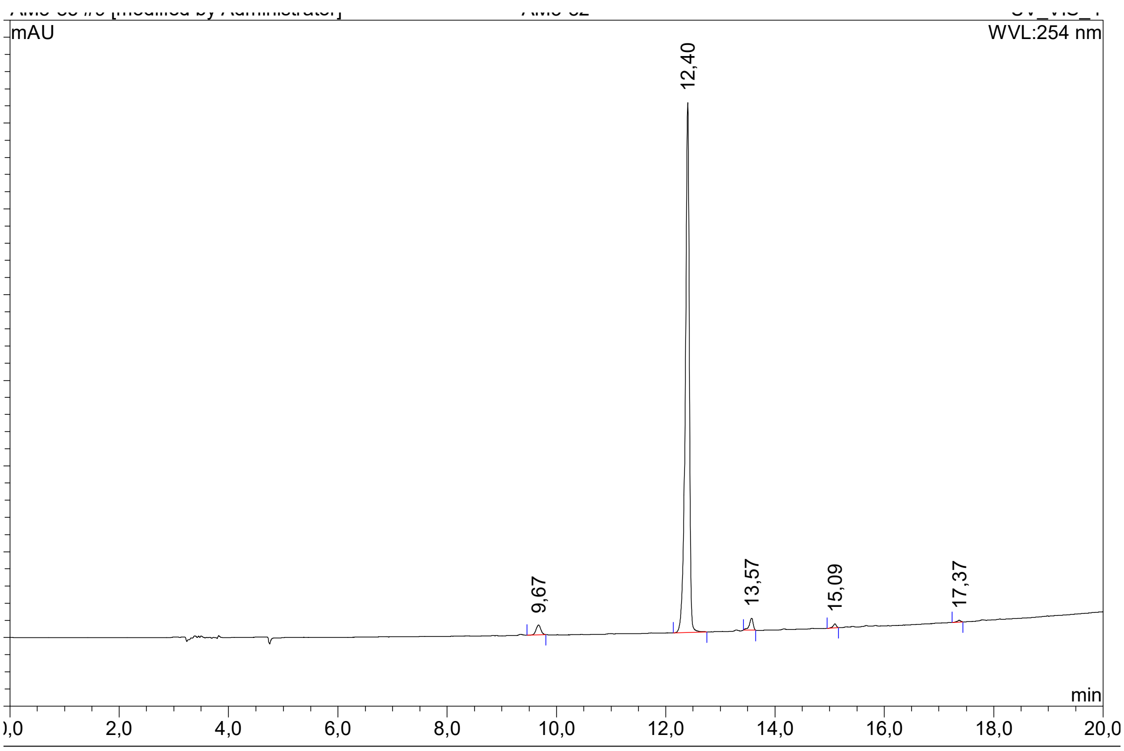

Supplementary Figure 3. NMR analyses of but-3-yn-1-yl methyl succinate (**5**)

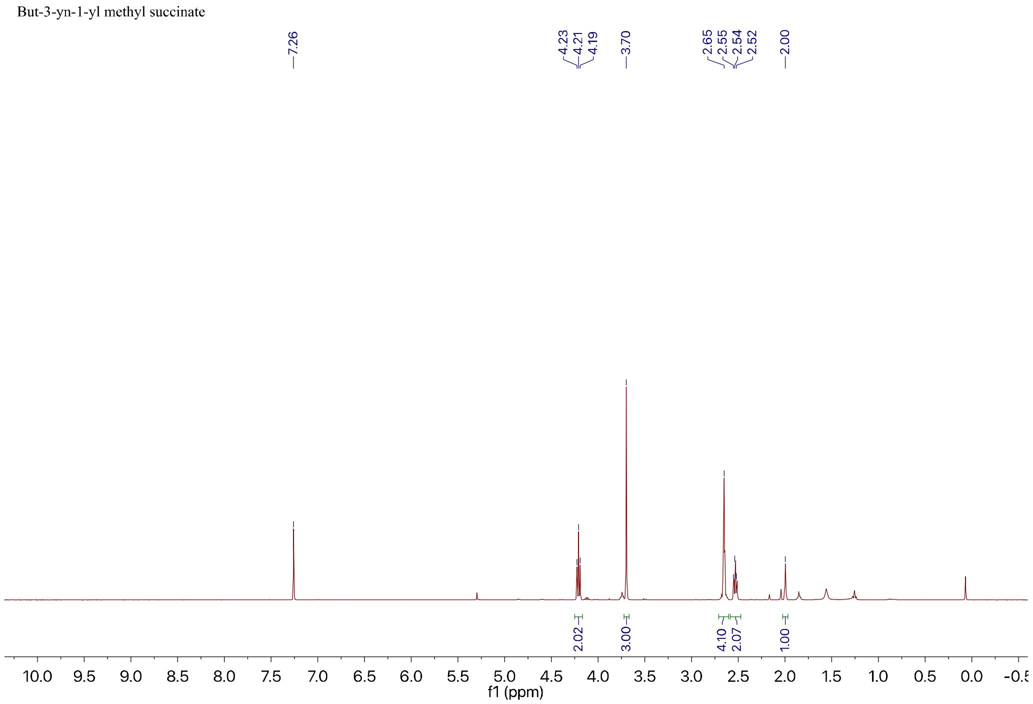

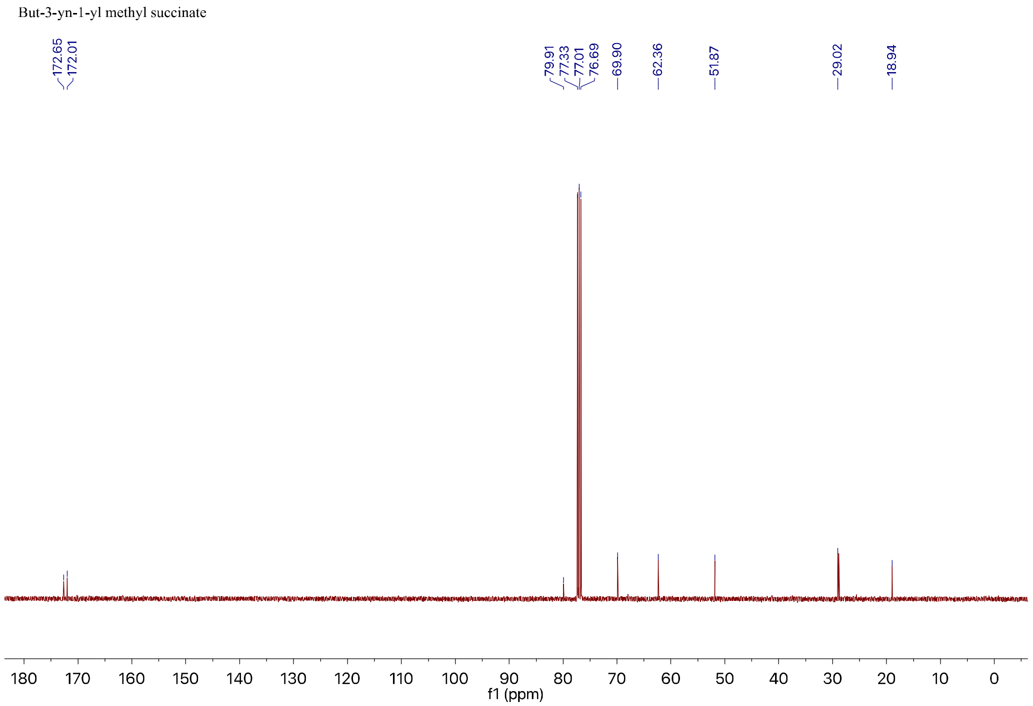

Supplementary Figure 4. HPLC analysis of compound but-3-yn-1-yl methyl succinate (**5**)

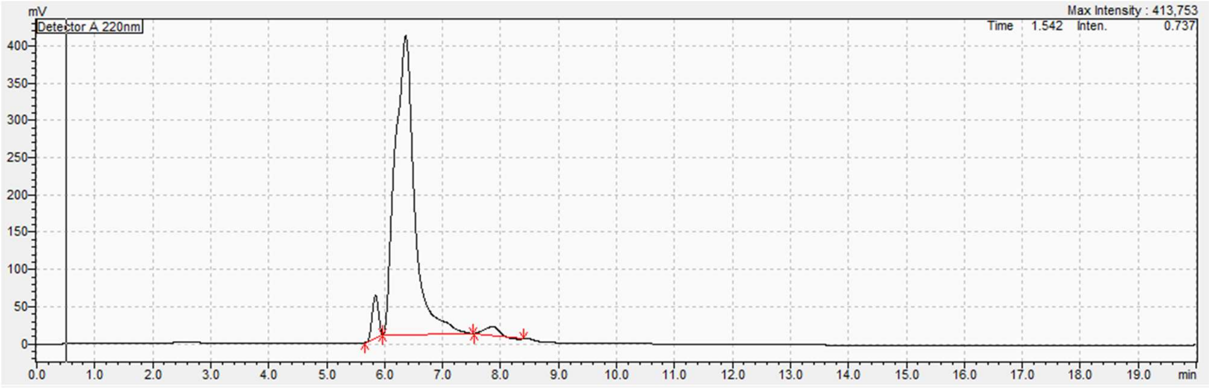

Supplementary Figure 5. NMR analyses of compound (*E*)-4-(but-3-yn-1-yloxy)-4-oxobut-2-enoic acid (**7**)

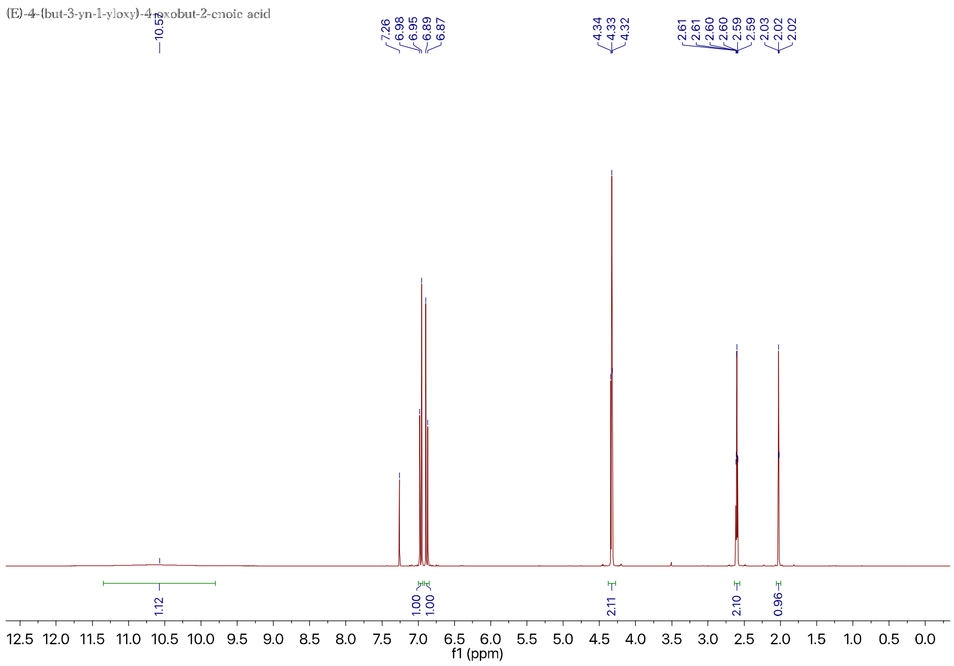

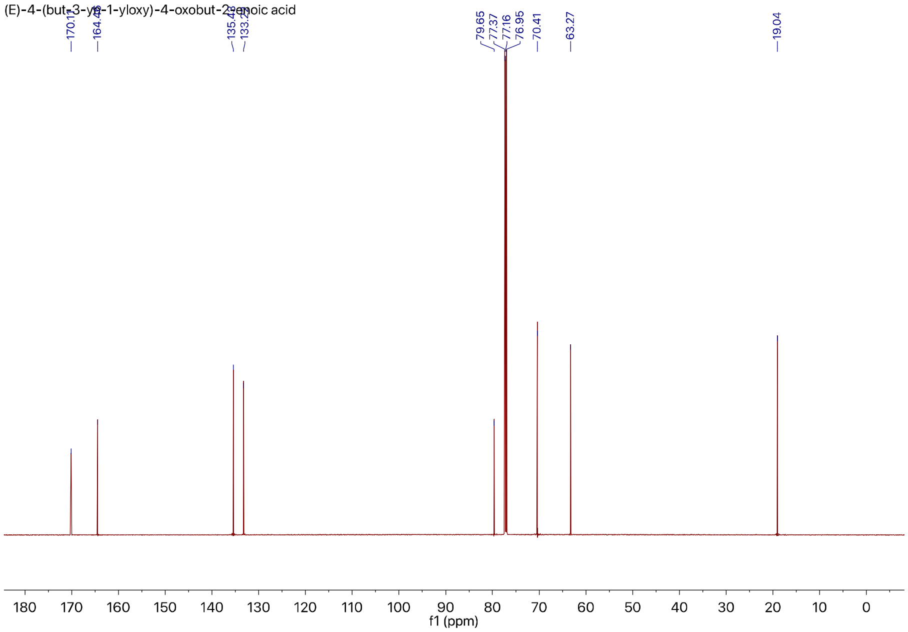

Supplementary Figure 6. HPLC analysis of compound (*E*)-4-(but-3-yn-1-yloxy)-4-oxobut-2-enoic acid (**7**)

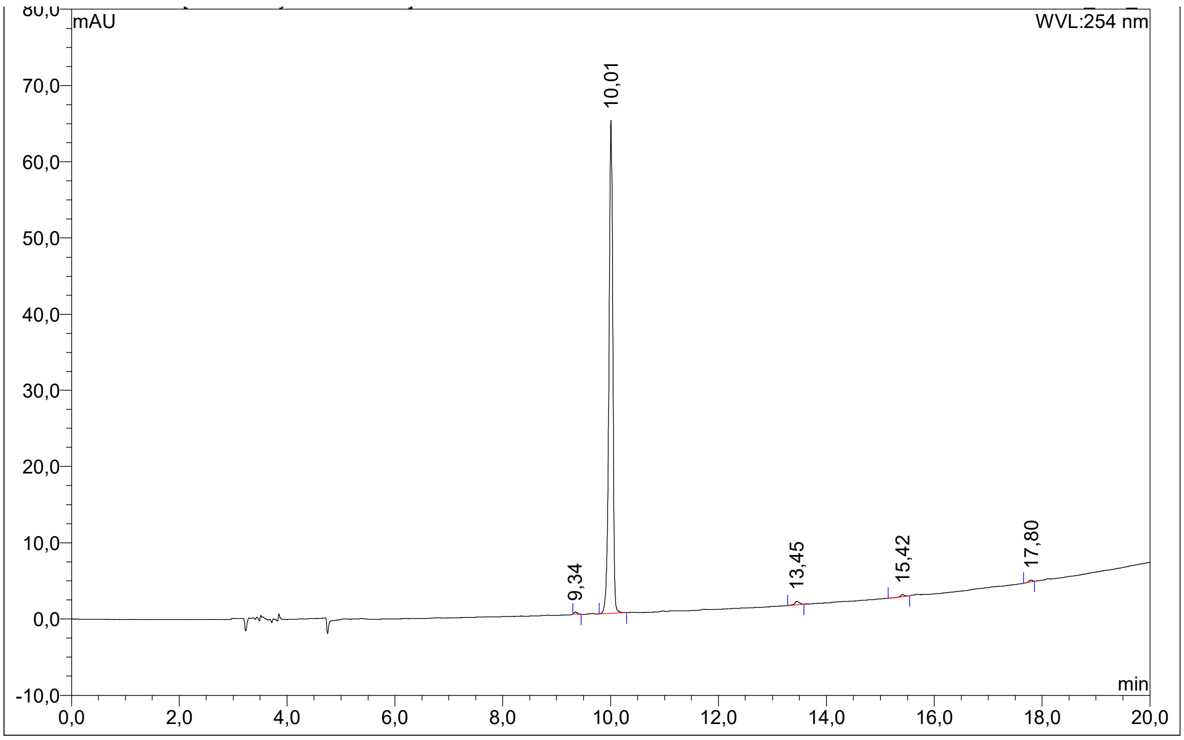

Python script for image analysis

The Python script below is designed to analyses the CZI image type in regards to fluorescence intensity. Each pixel intensity of the Alexa488 that is also positive for DAPI is taken into account. The focus on the nucleus prevents artefacts and ensures higher precision since both the DMFU- and MMFU-probes show a high degree of nuclear localization.

Python 3.0 Script:

```
import os
import czifile
import numpy
import matplotlib.pyplot as plt
import pandas as pd

#specifies the path to the picture folder. The folder should only contain 4 czi files
path = r"P:\Data\Cellculture\Cell_fluorescence\20210510_DMFU-Probe_DMFU_competition\triplicate_1_20x"
os.chdir(path=path)
#creates a list of all pictures in the folder
pictures = os.listdir()
#creates an empty dictionary and an empty pandas dataframe
box_dic = {}[3]
df = pd.DataFrame()
#Main function, that loops for each picture
for i, file in enumerate(pictures):
    j = 3 – i
    #opens the czi image and picks all channels and all pixels
    array = czifile.imread(pictures[j])
    #picks the dapi and dmfu channel as well as all the pixels in x and y for those two channels
    array1 = array[0, 0, :, 0, 0, :, :, 0]
    #split the channels in dapi and dmfu, devide by 60000 (or another number that is higher than the most intense
pixel) to get all the values between 0 and 1 the multiply by 265
    array1_dapi = (array1[1, :, :] /60000) *265
    array1_dmfu = (array1[0, :, :] /60000) *265
    #produce images from those arrays with uint8 type that cv2 can read
    img_dapi = array1_dapi.astype(numpy.uint8)
    img_dmfu = array1_dmfu.astype(numpy.uint8)
    #Analysis:
    #For easier analysis, flattens the 2D numpy array on to one dimention
    ana_dmfu = img_dmfu.flatten(order="C")
    ana_dapi = img_dapi.flatten(order="C")
    #Sets a Dapi threshold to identify the nucleus to prevent artefacts. Only the intensity of the DMFU probe in the
nucleus is analysed. To mesure all pixels, set the dapi threshold to 0
    dapi_threshold = 20
    mask = (ana_dapi > dapi_threshold)
    analysis = ana_dmfu[mask]
    #This tool can be used to check the dapi Mask. Do not use it in the final analysis run.
    ##plt.imshow((img_dapi) > dapi_threshold)
    ##plt.show()
    #Creates labels for the figures. Those labels need addaption for your experimental conditions. Each label contains
the experimental condition, the intensity avaerage and standard diviation
    labels = ["no DMFU", "30uM DMFU", "60uM DMFU", "90uM DMFU"]
    label = (labels[j] + "(" + str(round(numpy.average(analysis))) + " +/- " + str(round(numpy.std(analysis))) + ")")
```

```
    #Create a figure with 2 rows 4. And places the labeld pictures in each column in the first row.
    plt.subplot(2, 4, 1 + j)
    plt.title(label)
    plt.imshow(img_dmfu, vmin=0, vmax=140, cmap="inferno")
    plt.yticks([])
    plt.xticks([])
    # Boxplot analysis
    # Uses the earlier created dictionary and places the pixel intensity analysis to each label
    box_dic[labels[j]] = analysis
    #Creats the boxplot for the figure in the second row
    plt.subplot(212)
    labels_2, data = [*zip(*box_dic.items())]
    plt.boxplot(data, vert=False)
    plt.yticks(range(1, len(labels_2) + 1), labels_2)
    plt.xlabel("Intensity")
    #Exports the data to an excel file for further statistical analysis.
   df[labels[j]] = pd.Series(analysis)
   df.to_excel("results.xlsx", index=False)
#Shows an image of the used Pictures as well as a proliminary boxplot analysis
plt.tight_layout()
plt.show()
```

References

1. A. B. Lutjen, M. A. Quirk, A. M. Barbera and E. M. Kolonko, Synthesis of (E)-cinnamyl ester derivatives via a greener Steglich esterification, Bioorg. Med. Chem. 26 (2018), 5291-5298.

2. O. D. Ekici, Z. Z. Li, A. J. Campbell, K. E. James, J. L. Asgian, J. Mikolajczyk, G. S. Salvesen, R. Ganesan, S. Jelakovic, M. G. Grutter and J. C. Powers, Design, synthesis, and evaluation of aza-peptide Michael acceptors as selective and potent inhibitors of caspases-2, -3, -6, -7, -8, -9, and -10, J. Med. Chem. 49 (2006) 5728-5749.
